# Supplementary material for: Are carbon emissions trading and green financial instruments synergistic? -Comprehensive quantitative research based on content analysis
Source: PLoS One. 2024 Mar 7;19(3):e0298601. doi: 10.1371/journal.pone.0298601 (PMC10919601; doi:10.1371/journal.pone.0298601)
Supplement: S1 Data — (ZIP) [file pone.0298601.s001.zip › Data/地级市Z_F工作报告.zip!/╜¡╦╒/─╧═¿╩╨/└┤╫╘╣1⁄2╓┌║┼í╛┬φ┐╦╩2╛▌═°í┐_2015.docx]

各位代表：

　　现在，我代表市人民政府向大会作工作报告，请予审议，并请市政协委员和其他列席人员提出意见。

　　一、2014年工作回顾

　　过去的一年，我们在省委、省政府和市委的正确领导下，全面贯彻党的十八大和十八届三中四中全会精神，认真落实习近平总书记系列重要讲话精神和对江苏工作的要求，坚持稳中求进、改革创新，推进“八项工程、八个领先”，抓好十项举措、百项任务，较好完成全年工作目标。预计，地区生产总值5653亿元，增长10.5%；财政总收入1403.9亿元，一般公共预算收入550亿元、增长13.2%；固定资产投资3896亿元，增长18.1%；社会消费品零售总额2153亿元，增长11.8%；外贸进出口总额316亿美元，增长6.2%；注册外资实际到账25.8亿美元；城镇居民人均可支配收入33700元，农村居民人均可支配收入15850元，分别增长10%和11%；各项存款余额8508亿元，居民储蓄存款余额4624亿元、增长11.4%；居民消费价格涨幅2.1%；城镇登记失业率1.97%；全社会研发投入占GDP比重2.48%；完成省下达节能减排任务。

　　（一）经济发展继续稳中有进。以调促进、以进促稳，全市经济在加快转型升级中保持稳定健康发展。新增长点培育成效显现。制定稳定外资外贸、引进总部企业和支持海洋经济、文化产业、旅游业、养老服务业、楼宇经济发展等30多项政策意见，现代物流、服务外包等产业较快增长，通用航空、智慧建筑、机器人、创意设计、供应链管理、健康休闲等新兴业态加快培育。“智慧南通”建设快速推进，宽带IP城域网出口带宽达1324G，已落户智慧产业项目86个。产业结构调整稳步推进。工业六大主导产业实现产值1.1万亿元，33个市级以上特色产业基地提质增效，6家船企进入工信部行业规范条件企业名单，家纺电子商务、分布式光伏发电蓬勃发展。新兴产业“310”工程深入实施，产值占规模以上工业比重提高3.1个百分点，海洋工程、智能装备、节能环保等产业产值保持25%以上增长。建筑业施工总产值达5800亿元。服务业增加值占GDP比重提高1个百分点，50家市级以上集聚区营业收入增长23.9%，商务、科技和文化、旅游等服务业加快发展，狼山风景区成为省级旅游度假区。完成第三次经济普查。创新体系建设力度加大。大中型工业企业和规模以上高新技术企业研发机构建有率达94.9%，新增国家级企业技术中心1家、省级研发机构71家，高新技术企业330家。新建“三创”载体62万平方米，南通高新区、产业技术研究院、创源科技园等创新平台产业培育取得突破。获批全国质量强市示范城市。专利和知识产权工作成效明显。新引进国家“千人计划”人才28名、总数达107名，江海英才国际创业大赛10个获奖项目落户南通。新增创投、股权投资机构34家、总数达106家。

　　（二）全面深化改革开局良好。抢抓改革政策“窗口期”，积极争取国家级陆海统筹发展综合改革试点，全面展开各项改革，形成50多项改革意见和方案。经济体制改革加快实施。国资国企改革有序推进，与中航工业集团展开战略合作。民营经济转型升级“五项工程”深入实施，民资准入领域和门槛放宽，新增私营企业1.8万家，18家企业入选全省百强民企。成为国家海域综合管理创新示范市，陆海统筹土地管理创新方案纳入省节约集约用地“双提升”计划并获国土部批准，农村土地确权登记、产权制度等改革试点稳步推进。政府专项资金整合使用、地方政府性债务规范管理、风险防范得到加强，“营改增”等减轻和公平企业税负的政策有效落实。债券融资232亿元，新增上市企业2家、“新三板”等场外市场挂牌企业16家，跨境结算、离岸金融、融资租赁取得突破，银行业金融机构增至39家。行政体制改革持续深化。形成“4个5”简政放权、转变职能制度体系。制定健全并公布“5张清单”，取消、整合、下放审批事项147项，全面取消非行政许可审批事项，建立行政审批责任追究、行政权力清单管理等制度。建设项目并联审批、竣工联合验收机制基本形成，工商注册登记便利化改革全面实施。社会体制改革有序有力。统一城乡居民户口迁移准入政策。高职教育改革发展、中考改革试点和管办评新机制探索取得进展。县级公立医院医药价格综合改革全面实施，新一轮医改试点积极推进，城镇职工医保制度统筹、城乡居民医保制度并轨启动。推动报业、广电传媒集团组建。抓好市级17个政府购买服务试点项目。

　　（三）江海联动开发开放扎实推进。抢抓优化区域发展格局机遇，加快融入开发开放新布局。项目建设取得新突破。沪通长江大桥开工并快速建设，通洋高速一期、通州湾快速通道即将通车，宁启铁路复线电气化改造主体工程完成，宁启铁路二期开工。长江-12.5米深水航道一期完工，洋口港区、吕四港区码头航道建设有序实施，通州湾港区总体规划报批取得重要进展，通吕运河航道提升和东灶新河等江海河联运项目快速推进。列入省沿海开发五年推进计划的58个重大项目完成投资555亿元。沿海科教人才资源加速集聚。对接上海自贸区和长江经济带初显成效。积极复制上海自贸区体制机制创新经验，组织服务外包、贸易金融等专题招商，承接50多个“溢出”项目。在南通综保区实施工商注册登记“先照后证”和10项通关便利化措施，开展跨境贸易电子商务试点。贯彻国务院“长江经济带发展指导意见”，通州湾江海联动开发示范区创建积极推进，与武汉建立战略合作关系。引进来与走出去互动并进。外资结构优化，服务业利用外资比重提高6.4个百分点。内外贸一体化加快推进，30家企业入选省级重点出口品牌。获批省“走出去”先行先试试点城市，新批境外投资项目78个。举办中国中小企业节、南通江海国际博览会、台商产业转型升级峰会。中小企业示范园、两岸文创园区等通台合作项目落户。载体和平台功能增强。国家级、省级开发园区创新转型发展成效加速显现，“区中园”更具特色。跨国跨江合作园区功能进一步完善，中奥合作中心建成开放，中意海安生态园落户。洋口港区、吕四港区一类口岸开放和如皋港区保税物流中心获批，电子口岸关检港联动等5个系统上线运行，实现“三个一”联合通关和加工贸易无纸化通关。叠石桥市场采购贸易方式试点积极推进。纺织原料、油品交易和红酒展示展销等平台投入营运。

　　（四）新型城镇化和城乡发展一体化取得积极进展。启动新型城镇化试点，城乡五级空间体系积极构建。中心城市竞争力增强。253个城建项目完成投资310亿元，“一环一轴”快速路网基本贯通。新城区20个项目竣工或主体封顶，市北新城一批功能性项目快速推进，南部新城产城融合度显著提升，通州区重点片区功能加快完善。星岛水岸区域形象初步展现，通吕运河绿廊启动段工程竣工。西寺保护修缮、南公园改造完成，唐闸等历史文化保护项目有序推进。国家棚户区改造专项贷款到位56亿元，获得补助4.52亿元。重点城镇建设升温。全市城镇化率达61%。5个县（市）城改造建设势头良好，新一轮城市总体规划修编全面完成。10个镇入选全国重点镇。19个市级中心镇新开工77个超亿元产业项目、319个基础设施和民生改善项目，基本公共服务资源配置得到加强，行政管理体制改革试点有序推进。农业现代化进程加快。粮食生产实现“十一连增”。“三资”农业项目投资250亿元。新增“全托管”经营主体367个、市级以上龙头企业50家、省级示范家庭农场10家，122家农民合作社成为省级以上示范社。新（扩）建规模农（渔）业园区15个。完成水利建设投入25.8亿元。新建高标准农田24万亩。农业机械化水平持续提高。

　　（五）生态文明建设力度加大。实施生态文明建设三年行动计划，生态环境持续改善。生态创建扎实开展。国家生态市建设通过环保部考核验收。实施70个循环经济和248个减排项目，南通开发区获批国家循环化改造示范试点园区和国家生态工业示范园区，中水回用项目投入运行，4个开发园区建成省级生态园区。生态红线区域保护有力推进。连续23年实现耕地占补平衡。全市新增造林18.5万亩，市区新增绿地770公顷。环境治理持续加强。环保执法亮剑行动有力实施。燃煤锅炉改造、扬尘管控等大气污染防治措施全面落实，PM2.5平均浓度降幅和空气质量良好天数领先全省。化工园区整治和落后产能淘汰积极推进。城乡河道、重点水系治理力度加大，实施了“河长制”。市区日常饮用水源和应急水源“双水”保供体系基本建成，获批国家水生态文明城市建设试点。市区完成205个市容环境综合整治项目。村庄环境整治通过省级全域性考核验收，获省人居环境范例奖。制度创新取得突破。严格化学品生产环保准入管理，初步建立陆海生态环境一体化管理、水环境区域生态补偿、排污权有偿使用和交易、领导干部资源环境责任审计等制度，开展环境信用评级，落实绿色信贷、差别水电价等环境经济政策。

　　（六）民生幸福水平持续提升。一般公共预算支出中民生投入比重达57.2%，10件48项为民办实事项目全面完成（见附件一）。就业保障工作不断强化。新增就业9.1万人，扶持创业1.2万人。出台市区征地补偿和被征地农民社会保障办法，企业退休人员养老金、城乡居民基础养老金、城镇居民医保筹资、城乡低保等标准继续提高，大病保险制度实现城乡全覆盖。社会救助形成制度化安排，扶贫攻坚机制加快健全。公共服务能力稳步提高。省市共建教育现代化市级示范区启动，学前教育普惠园、省优园占比进一步提高，市区义务教育全面推行联合办学等均衡发展措施，5所高校升格、更名。举办首届公共文化展示月活动，15部文艺作品获全国、全省“五个一”工程奖，唐闸近代工业遗存“申遗”进入国家评审阶段。基层医疗卫生服务能力持续加强，城市医疗资源优化配置继续推进，与上海等地名院名医开展深度合作。市区安置动迁户1.3万户，公共租赁房和廉租房并轨运行。每千名老人拥有养老床位超过29张，基本形成“9073”养老服务格局。“菜篮子”工程取得新进展。南通籍健儿在南京青奥会、仁川亚运会上获得5枚金牌。单独两孩政策全面实施。社会治理体系逐步完善。社会综合服务管理平台实现县（市）区全覆盖，矛盾纠纷大调解体系不断完善，稳评、安评、环评联动实施。政社互动、街坊邻里自治等深入推进，全市城乡和谐社区省级达标率分别达85%、78%，法治环境公众满意率居全省前列。“五大专项行动”扎实开展。应急管理反应机制进一步规范健全，食品药品监管和民族、宗教等工作不断加强。军政军民关系更加密切。群众性精神文明创建取得丰硕成果，蝉联全国文明城市称号。

　　各位代表，我们以开展党的群众路线教育实践活动为抓手，加强政府建设，完成机构改革任务。服务效能实现新提升。深化行政审批“三集中、三到位”，政务服务综合标准化试点扎实开展。整合建立“12345”政府公共服务平台。加强和规范政府督查工作，市政府100项年度重点工作全面完成。依法行政取得新进展。落实“三重一大”等集体决策制度，强化行政权力网上公开透明运行，深化城管执法体制改革，加强行政复议等工作。拓展政务公开范围。自觉接受市人大及其常委会的法律监督、工作监督和市政协的民主监督，认真办好市人大关于“绿色南通”建设的议案和市政协关于推进农村土地流转、构筑技能人才高地的建议案，完成人大代表建议、政协提案办理任务。工作作风得到新改善。严格执行中央八项规定和省、市委十项规定，以“钉钉子”精神抓好教育实践活动整改和系列专项治理行动，落实“三清示范区”建设举措，政府系统“四风”问题得到有效整治。市级机关公用经费削减6.9%。行政监察、审计监督力度加大。

　　各位代表，过去一年，各项成绩的取得，是省委、省政府和市委正确领导，市人大、市政协监督支持，全市人民团结一致、开拓创新、艰苦奋斗的结果。我代表市人民政府，向全市人民，向人大代表、政协委员和离退休老同志，向市各民主党派、工商联、各人民团体和社会各界人士，向驻通人民解放军、武警官兵和公安干警以及部省驻通单位，向关心和支持南通发展的海内外朋友，致以崇高的敬意和衷心的感谢！

　　各位代表，我们也清醒认识到发展中的问题。经济结构调整任务艰巨，新兴增长点培育不快，服务业占比还不高，经济增长动力不足，部分企业生产经营困难。有的领域改革攻坚力度还不大，一些举措落地见效还不快。破除城乡二元结构仍面临许多难题，城镇化质量有待提高。生态持续改善的压力加大，环保基础设施和能力建设仍显滞后。民生保障还有一些薄弱环节，居民收入增长空间不足、增速放缓。社会治理遇到不少新情况新挑战，各种不稳定因素相互交织，安全生产事故时有发生。同时，政府职能转变还不到位，依法行政能力还不强，思维定势、行为惯性、路径依赖没有完全破除，反“四风”转作风的长效机制有待完善，反腐倡廉仍需加强。我们一定采取有效措施，认真加以解决。

　　二、2015年目标任务

　　今年是全面深化改革、全面推进依法治国、全面完成“十二五”规划的重要一年。世界经济格局深度调整，在总体缓慢复苏中呈现分化、波动、变革的特点；我国经济发展进入新常态，产生一系列趋势性变化，既有韧性好、潜力足、回旋空间大的有利条件，也有增长速度换挡、发展方式转变、经济结构调整、发展动力转换的阵痛。我市既具备多重国家战略叠加的比较优势，又面临化解经济下行压力、跨越中等收入阶段等诸多难题。我们必须坚定信心、抢抓机遇，坚持发展、主动作为，解放思想、鼓励首创，推动各项工作同步提升，开创“两个率先”新局面。

　　今年政府工作的总体要求是：全面贯彻党的十八大和十八届三中四中全会精神，以邓小平理论、“三个代表”重要思想、科学发展观为指导，深入落实习近平总书记系列重要讲话精神和对江苏工作的最新要求，按照中央、省委省政府和市委的部署，主动把握和积极适应新常态，坚持稳中求进工作总基调，坚持以提高经济发展质量和效益为中心，与时俱进推进“八项工程、八个领先”，以创建陆海统筹发展综合配套改革试验区为突破口，深化改革开放，着力创新驱动，保障改善民生，强化风险防控，推进依法行政，促进经济平稳健康发展、社会和谐稳定。

　　今年经济社会发展的主要预期目标是：地区生产总值增长9.5%左右；一般公共预算收入增长10%；固定资产投资增长16%左右；社会消费品零售总额增长13%左右；进出口总额保持增长；城乡居民收入增长与经济增长同步；居民消费价格涨幅3%左右；城镇登记失业率4%以内；全社会研发投入占GDP比重达到2.5%；万元地区生产总值能耗下降、主要污染物减排完成省下达任务。

　　做好今年工作，我们将紧紧围绕经济强、百姓富、环境美、社会文明程度高，牢牢把握“五个迈上新台阶”的重点任务，着力拉长补齐短板，争当苏中新一轮发展“领头雁”、建设长三角北翼经济中心。突出提质，坚定不移转方式调结构。更大力度推进转型升级、创新驱动、改革开放，推动发展方式转向质量效率型集约增长、结构调整转向优化存量与做优增量并举、发展动力转向新的增长点。着力扩量，保持稳增长和调结构平衡。激发各种所有制经济和市场主体竞相发展，科学运用政策创新等手段，千方百计稳增长，推动发展在中高速增长中迈向中高端水平，做到调速不减势、换挡不失速、量增质更优。持续增效，强化“四化”同步提升协调发展水平。更好发挥市场机制作用释放红利，更多依靠产业化创新挖掘增长潜力，更加注重产城融合增强综合功能，加快江海联动、城乡统筹发展进程，实现经济效益、社会效益和生态效益齐头并进。全力惠民，坚持以人为本推动社会全面进步。积极满足群众个性化、多样化需求，加快构建社会共建、共享、共治新格局，让改革发展成果更公平惠及广大人民。

　　工作中，着重抓好十个方面：

　　（一）突出加快转型升级，进一步保持经济持续稳定增长。坚持转调并举，推动“进”和“稳”有机统一、相互促进。

　　调优主导产业。加快制造业升级。促进六大“千亿级”产业稳中提质，加大技改投入，打造领军企业，加快化解过剩产能、淘汰落后产能。做大绿色、智慧建筑等特色产业。提高服务业发展水平。狠抓项目投入、企业培育，做大做强“7+3”重点行业，服务业增加值占GDP比重超过43%。依托创新驱动、人才引领，加快生产性服务业和新兴服务业发展。促进优势产业集聚。实施重点产业补链计划，强化特色产业基地和集群建设，提高海工船舶、电子、家纺等竞争力。推进现代物流和科技、商务等服务业集聚区提升，增强要素吸附、产业支撑和辐射带动能力。

　　壮大新兴业态。推动新兴“种子”产业集聚。突出“3+4”重点领域，加快推进“310”工程，新兴产业产值超过4500亿元。推动新能源、新材料等产业提档升级，发展机器人、3D打印等智能制造。推进通用航空特色园区建设，布局生物医药、医疗器械等生命科技产业。发展服务新业态。加快培育平台经济，推进供应链管理、电子商务、研发设计、检验检测等重点平台建设。推动服务外包专业化、规模化发展。壮大楼宇经济、总部经济和后台服务产业。推进“智慧南通”建设。落实顶层规划，加快信息基础设施布局，宽带IP城域网出口带宽达2000G。以智慧城市建设带动智慧产业发展，培育大数据、云计算、移动互联网、互联网金融、物联网等新增长点，打造互联网商业模式示范企业和智慧产业园区，促进互联网经济与实体经济嫁接融合。

　　提升现代农业。构建现代农业产业体系。稳定粮食生产，抓好“菜篮子”工程。推进国家现代农业示范区建设，实施主导产业“6231”工程和特色产业“双百行动计划”，加快农业标准化、专业化、品牌化、信息化进程。培育新型农业经营主体。加快发展合作组织、家庭农场和专业大户，新增市级以上龙头企业40家、“全托管”示范点300个。引导土地经营权有序流转，推动适度规模经营。培养有文化、懂技术、会经营的职业农民。强化农业物质装备和科技进步。深化农业综合开发，实施土地综合整治示范工程，新建高标准农田20万亩。创建国家农业科技园区，提升农技推广和农业机械化水平，完善农业公共服务体系。

　　强化“三驾马车”带动。发挥投资关键作用。保持有效投入强度，围绕稳增长、调结构、补短板、惠民生，鼓励各类资本投入。重抓项目推进，实施总投资超5000亿元的160个市级重大项目。发挥外贸支撑作用。着力稳定和扩大出口，改革贸易方式，强化市场开拓，加大新增长点培育和特色基地建设力度。拓展先进技术、产品进口，壮大服务贸易，带动进出口、内外贸协调发展。发挥消费基础作用。多措并举引导消费，丰富商业和消费业态。打造“畅游南通”品牌，加强旅游项目建设管理，培育一批生态旅游示范区和乡村旅游集聚区。推进一批智慧应用项目，推动网络购物与商贸流通、快递业融合发展，争创国家信息消费综合试点城市。挖掘健康、养老、体育等消费潜力。

　　（二）着力强化创新驱动，进一步建设创新型城市。抓住产业、企业、载体、人才、环境等关键要素，深入推进国家创新型试点城市建设，促进“四个对接”，让创新成为驱动发展新引擎。

　　增强企业创新能力。发挥企业创新主体作用，加大“五自”企业培育力度，力争高新技术企业总数达700家、产值占规模以上工业比重达45%。健全技术创新市场导向机制，新增省级以上研发机构35家。深化全国质量强市示范城市和知名品牌示范区建设，增强优势产业技术标准话语权。全面落实鼓励创新的政策，支持企业开展产业核心技术攻关，引导企业加大研发投入。

　　完善区域特色创新体系。推动科技进步和全面创新，促进创意创新创业创优融合。深化协同创新。实施300项产学研合作项目。支持重点园区、乡镇与知名院校、科研机构共建协同创新基地、研发平台，加快创新成果转化。围绕重点产业链部署创新链，实施技术创新集成支持。优化创新载体。增强南通高新区、产业技术研究院等高端平台引领功能，完善新技术研发应用和产业化体系。争取省产业技术研究院在通设立机构。新建“三创”载体50万平方米。加强公共技术服务平台、重点实验室、中试基地等建设。完善创新环境。强化政策、制度等综合保障，一般公共预算收入的4%用于科技人才发展。推进省级科技金融合作创新示范区建设，实现县（市）区和重点园区创投机构全覆盖，打造一批集聚创投机构的财富中心。深化国家知识产权示范城市建设。

　　加快创新创业人才集聚。坚持引进、培养、激励并举，提升人力资本素质，激发各类人才创新创造活力。拓宽引才用才渠道，用好江海英才创业周、国际创业大赛等平台，集聚一批引领产业发展的优质人才项目，新增国家“千人计划”人才20名、省“双创”人才30名。重视技能型、实用型人才培养，加强企业家队伍建设。着力打造懂市场、懂政策、懂操作的服务团队。

　　（三）切实抓好重点领域改革，进一步释放内生动力活力。以陆海统筹发展综合配套改革为重点，加快改革举措落地见效。

　　促进多种所有制经济竞相发展。推动民营经济转型升级。大力发展民营经济，支持民营企业创新发展，深化特许经营、政府购买服务等改革，鼓励民资进入公共服务、资源环境、基础设施等领域，做强民营经济特色板块。深化国资国企改革。加强国有资产和资源整合，深入推进国有企业分类改革，健全以管资本为主的国资监管制度，提高国有资产经营管理水平。推进多种所有制经济融合。鼓励国有、集体、非公等资本交叉持股，支持本土企业以股权转让、增资扩股、联合投资等方式引进战略投资者。

　　完善现代市场体系。以互联网思维，推动有形市场与无形市场相互融合、商品市场与要素市场相互促进。健全要素市场，深化公共资源和综合产权交易改革，构建城乡统一的建设用地市场，强化海域资源市场化配置，培育技术交易、服务和成果转让等综合性技术市场，建好新的人力资源市场。有序实施价格改革。

　　深化财税金融体制改革。构建全口径预算管理体系，加强政府专项资金绩效管理，强化地方政府性债务规范管理，改革财政支持经济发展方式。积极发展银行业金融机构和金融租赁、信托、保理等新型金融机构。创新金融平台、工具、产品，扩大直接融资、贸易融资，推进企业上市和场外市场挂牌，形成区域金融特色。拓展境外融资、跨境贸易人民币结算和离岸业务、跨国公司资金集中运营等试点。落实定向减税、普遍性降费和降低企业融资成本等措施，更好支持中小微企业发展。

　　探索城乡发展一体化改革。开展新型城镇化试点，全面推进市级中心镇行政管理体制改革。有序实施农村土地征收、集体经营性建设用地入市、宅基地制度等改革，推动土地承包经营权确权登记，引导基层稳妥推进“三集中、三置换”。完善新型城镇化投融资机制，探索农村产权交易制度，发展新型农村金融组织。

　　加快社会和生态领域改革。深化教育领域综合改革，推进管办评机制创新，加快现代学校制度建设，促进办学主体多元化。深化医药卫生体制改革，推进公立医院综合改革，健全分级诊疗机制，鼓励社会力量办医。完善文化管理体制和文化产业经营机制。强化排污权交易、生态补偿等环境经济政策杠杆作用，开征扬尘排污费，探索环保准入清单管理制度。

　　（四）大力推进经济国际化，进一步增创开发开放新优势。坚持“六外”并举，增强在境内外配置资源、开拓市场的能力。

　　对接上海自贸区，增强开发园区功能。深入学习、接轨、配套、服务上海自贸区。加快承接“溢出”项目，推广投资贸易便利化、金融和服务业开放等体制创新经验，健全外商投资清单式审核、备案化管理制度，推进市场采购贸易方式和跨境贸易电子商务试点，完善国际贸易电子数据“单一窗口”模式。提升开发园区国际竞争力。推动南通开发区和综保区发挥开放创新引领作用，支持新升格国家级开发园区完善运行机制、提升综合功能。促进省级开发园区强化特色产业基地和“区中园”建设，力争新增1-2家国家级开发园区。强化开放平台功能支撑。推动重点开发园区深化大宗商品交易、物流通关、金融服务等平台建设，探索构建重点商品区域性、流域性指数发布平台。拓展电子口岸功能，加快融入长三角和长江流域通关一体化体系。支持有条件的区域布局海关特殊监管区，推动兴东机场加快口岸开放。

　　强化招商选资，提升引进来质量。坚持多种资本并举，强化产业链、专业化和政企联动招商，注册外资实际到账25亿美元，引进超亿美元外资项目10个、超亿元市外民资项目100个。主攻欧美、日韩、台湾等区域，加强新兴产业、服务业和新技术、新人才招引，更多引进融资租赁、总部企业和功能性机构。

　　参与“一带一路”建设，加快走出去步伐。抢抓机遇，拓展东盟、中南亚、非洲、中东欧等新兴市场，放大走出去先行先试效应。支持创建境外经贸合作区、境外产业集聚区，引导境外投资向并购优质资产、知名品牌、营销网络和综合开发转变。加强外事工作，深化与重点区域、国际友城的互利合作。

　　（五）深入实施陆海统筹江海联动，进一步放大特色发展融合发展成效。对接国家战略，放大独特优势，强化对上海的配套服务，在更高层次、更广领域推进陆海统筹江海联动，实现自身发展与服务区域发展共同提升。

　　深度融入长江经济带发展。围绕打造长江经济带北翼桥头堡，完善江海河、公铁空多式联运体系。加快通海港区、如皋港区建设，推进洋口港区15万吨级、吕四港区10万吨级航道工程和通州湾腰沙开发。加快沪通铁路、宁启铁路二期建设，开工海启高速。推进九圩港复线船闸、通扬运河绕城段改造等江海河联运项目。做好通苏嘉城际铁路、锡通高速、崇海大桥、长江北支航道整治前期工作。加强与上海的港口、机场等合作，深化与武汉等长江中上游重点城市合作。强化联盟发展，加快通州湾江海联动开发示范区建设。

　　加快沿海港产城融合发展。落实省沿海开发“六大行动”方案，强化规划建设统筹，促进错位发展。重点支持洋口港区建设液化品物流和高分子新材料产业基地，通州湾港区建设高端装备、海洋产业和综合物流基地，海门东灶港区建设建材物流和重型装备产业基地，吕四港区建设粮油物流和船舶海工产业基地以及现代渔港经济区。大力发展海洋经济，培育海洋新兴产业和滨海旅游业。加快沿海城镇建设，形成滨海城市特色。

　　推动沿江转型发展。健全沿江区域产业梯度转移升级引导机制，推进岸线整合调整，优化港口、产业、功能布局。支持苏通、锡通科技产业园和上海市北高新（南通）科技城等合作园区，发展新产业、新城市、新人才，加快中奥苏通生态园、中意海安生态园建设。接受苏南现代化建设示范区辐射，推动苏通经济圈建设，加快基础设施、产业体系、政策环境等共建共享。

　　（六）完善中心城市发展体系，进一步提升综合竞争力。积极推进城市总体规划实施“多规合一”，彰显江海城市新魅力。

　　加强城市建设。推进总投资223亿元的282个重点城建工程，实现组团互联互通、功能显著提升、片区集聚发展。强化交通引领。推进长江路西延、机场大道等快速路工程，实施46个骨干路和一批支路项目，完善重点组团、重要节点交通连接体系。深化轨道交通建设前期工作。增强经济支撑。优先发展2.5产业、功能性服务业、文化创意等产业，做强一批特色产业园、总部经济园和主题楼宇、商业街区。加快片区开发。增强观音山新城配套功能，有序推进新城区东部商业金融文化集聚区和通沪大道沿线建设。以开发区能达商务区、苏通园区为重点，带动南部新城形象提升。围绕北大街商圈、物流园等重点板块，强化市北新城功能开发。完善通州区与主城区连接通道，促进东部新城功能片区繁荣。加快南通滨海园区新城建设，增强要素集聚能力。

　　提升城市品位。更加注重精致、细腻、整洁、有序，实施新一轮城市环境综合整治三年行动计划。推进城中村、老小区、危旧房整体改造和道路沿线综合改造，加强小区物业管理。抓好唐闸古镇核心区修建，有序实施历史文化保护项目。加快“两河两岸”棚户区域改造和配套功能建设，推动星岛水岸区域业态引进、交通优化。构建狼山风景区慢行系统，完善濠河景区功能。

　　优化城市服务。加快智慧城市建设，推动服务功能提升。加强公交首末站和换乘中心建设，增加公共场所和老小区停车位。加快一批综合性公园、专类园、小游园和道路绿化带建设，健全慢行步道、文体健身等设施。推进社区邻里中心建设，完善教育、医疗等公共服务网络。提升城市水电气服务能力，实施崇海水厂二期等29个公用事业项目。健全防洪排涝体系。

　　（七）扎实有序推动新型城镇化，进一步促进城乡发展一体化。突出以人为本的新型城镇化，提升城乡统筹发展水平。

　　有序推进农业转移人口市民化。鼓励各地探索符合实际的农业转移人口市民化路径，构建成本科学分担机制。深化户籍制度改革，提高城镇常住人口落户率，全面推行居住证制度。加大资源配置力度，推进就业、社保、教育、医疗、文化、住房等基本公共服务均等化。依法保障农业转移人口合法权益。

　　发挥重点城镇纽带作用。推动县（市）城、中心镇、特色镇协调发展，全市城镇化率提高1个百分点。加强规划指导，支持县（市）城增强对县域经济、社会、人口发展的承载力，提升集聚和配套功能。引导国家、省、市重点镇因地制宜、产城融合，推动设施完善、产业集聚、服务提升，加快向现代化小城市发展。放大资源优势，打造一批宜业宜居宜游的绿色智慧人文特色镇。

　　提升新农村发展水平。落实强农惠农富农政策，加大“三农”资金投入力度。优化镇村布局规划，打造一批留得住乡愁的特色村庄。加强农村基础设施改造建设，实施九圩港提水泵站等重点水利工程，推进一批干线公路项目和农村公路提档升级，积极发展镇村公交。实施拆坝建桥工程，疏通农村河道水系。巩固村庄环境整治成果，创建农村环境“四位一体”长效管理示范乡镇，确保“美丽乡村”试点全部通过省级验收。

　　（八）持续加强环境治理和保护，进一步提高生态文明建设水平。坚持源头严防、过程严管、后果严惩，深入推进“九大行动”， 启动生态文明建设示范区创建，全力守护蓝天净水。

　　推动绿色低碳循环发展。实行严格的环境准入标准、污染物排放标准和产品能耗限额标准，实施一批重点节能工程和减排项目。深化重点行业标准化整治和化工园区生态化创建，推动化工企业进园区。推进循环经济重大项目，建设一批循环化改造示范试点园区，力争省级以上开发区全部建成生态园区。完善能源、水、土地、海域节约集约利用制度，提高资源开发综合效益。

　　狠抓重点领域污染防治。打好大气污染防治持久战、攻坚战，加大“控煤、降尘、治企、管车、禁烧、联防”力度，切实减少污染排放，确保PM2.5平均浓度持续下降。疏堵结合加强水污染防治，完成市区水环境整治三年任务，争创国家级节水型城市。强化备用水源建设和水源地隐患整治，确保饮用水安全优质。推进农业面源、固体废弃物、餐厨废弃物和土壤污染防治。严格执行新环保法，深入开展环保执法亮剑行动，健全环境保护联动执法机制，完善企业环境行为信用评价制度。

　　加强生态保护建设。严格落实生态红线区域保护制度，强化十大类60个生态功能区和生物多样性保护，加快形成节约资源、保护环境的产业结构和国土空间开发格局。提升“绿色南通”建设水平，市区新增绿地800公顷，全市林木覆盖率提高1个百分点。构建陆海生态环境基础设施体系，加强涉海工程管理和海洋环境保护。深化基层生态创建，强化环保宣传教育手段创新。

　　（九）更好保障改善民生，进一步加大社会建设力度。突出保障基本民生、关注低收入群众，深入实施民生幸福工程，健全社会建设和公共服务体系，办好10件45项惠民实事（见附件二）。

　　促进就业创业富民增收。加大创业基地建设、小额担保等政策落实力度，促进大众创业、万众创新、群众就业。深入实施大学生就业促进计划和创业领航计划，统筹抓好农村劳动力和就业困难人员就业工作，新增就业8万人。健全工资集体协商机制。多渠道增加居民经营性、财产性收入，稳步提升转移性收入。壮大村级集体经济，促进农民增收。完善扶贫开发机制，确保农村年人均纯收入低于5000元的农户全部脱贫。

　　提高城乡社会保障水平。实施全民参保登记计划，提升社会保险扩面征缴质量。增强城镇职工医保统筹能力，健全城乡统筹的居民医保、居民养老保险制度，提高大病保险保障水平，逐步缩小城乡低保差距。优化保障性住房建设和供应，加快动迁安置房建设，多种途径满足群众差别化保障需求。支持社会力量兴办养老机构，鼓励“医养结合”，每千名老人拥有养老床位超过30张。完善社会救助体系，健全救助联动保障机制。积极发展妇女、儿童、青少年、残疾人、关心下一代、红十字、慈善等事业。

　　推进教育现代化市级示范区建设。完善学前教育公共服务体系，大力发展普惠性幼儿园。加快义务教育学校标准化、现代化建设，缩小区域、城乡、校际差距。扩大普通高中优质资源总量，推进特色多样化发展。促进职业教育产教融合，推动高等教育国际合作，提高服务经济社会发展能力。统筹抓好特殊教育、社会教育。加快建设“智慧教育”，促进优质教育资源开发与共享。

　　构建现代医疗卫生体系。推动城市优质医疗资源向薄弱区域扩展，支持县级医院提标升级，加强专科医院建设，夯实基层卫生服务基础。强化人才建设，培养一批全科医生。提高城乡医疗服务水平，构建和谐医患关系。加快“智慧卫生”建设，深化协同创新，加强与上海等地名院名医合作。推进计生服务标准化。

　　推动文化大发展大繁荣。培育先进文化、创新传统文化、扶持民间文化、引导流行文化，彰显江海特色文化魅力。发挥文化引领作用。把践行社会主义核心价值观和弘扬新时期南通精神有机结合，引领社会新思潮、凝聚发展正能量。加强诚信南通和公民道德建设。巩固文明城市常态化、长效化创建成果。加大文化惠民力度。加快构建现代公共文化、体育服务体系，强化服务设施布局和资源整合。加强书香南通建设。繁荣文化艺术生产。促进全民健身，提升竞技体育水平。加快文化产业发展。推进80个文化产业项目，文化产业增加值占GDP比重达到4.5%。推动传统媒体与新兴媒体、文化创意和设计服务与相关产业融合发展，鼓励文化企业创新，培育品牌示范园区。

　　（十）全面推进依法治市，进一步增强社会治理能力。深化法治南通建设，用法治思维和方式提升社会治理水平。

　　强化基层基础工作。加强基层民主和法治建设，健全民主选举、决策、管理和监督机制，增强依法自治能力。巩固村（社区）减负成果，完善基层综合服务管理平台，90%的城市社区、80%的农村社区达到省级和谐社区标准。推动社会组织改革和健康、有序发展，促进政社分开、政社互动，加强行业协会、商会和公益慈善、社区服务等社会组织的培育管理。

　　依法化解社会矛盾纠纷。健全排查预警等制度，深化“一综多专”大调解工作，强化人民调解、行政调解、司法调解联动，依法依规、及时就地化解矛盾纠纷。畅通利益诉求表达渠道，引导群众依法理性维权。完善社会稳定风险评估机制。推动信访工作纳入法治化轨道。强化法治宣传，完善公共法律服务体系。

　　推动平安法治融合发展。完善立体化、现代化社会治安防控体系，依法防范、严厉打击各类违法犯罪活动。实行最严格的安全生产、环境保护、食品药品安全监管措施，全面落实企业主体、部门监管和属地管理责任，全面推广化工企业设置“二道门”，确保人民生命、财产和健康安全。强化信息网络安全管理。推进军民融合深度发展，争创全国双拥模范城“六连冠”。加强国家安全、应急管理、民族、宗教、档案、保密等工作。

　　各位代表，今年也是谋划“十三五”发展的重要一年。我们将充分发扬民主，集中各方智慧，认真编制下一个五年规划，研究制定新一轮经济社会发展的目标、思路和举措，着力增创新常态下创新发展、科学发展、可持续发展优势。

　　三、建设法治政府和服务型政府

　　按照推进治理体系和治理能力现代化的要求，依法全面履行政府职能，加快建设人民满意的法治政府和服务型政府。

　　（一）深化简政放权、职能转变。加快构建“小政府、大社会、全服务”模式，做到法定职责必须为、法无授权不可为。加强清单管理。健全行政审批等“5张清单”动态调整机制，依法下放权力、减少事项、简化环节，提升行政服务效率。加强行政审批流程再造，扩大并联审批、网上审批。强化职能整合和基层服务能力建设，保障行政权力放得下、接得住、运转得好。推进政府事权规范化、法治化。优化服务体系。按照横向集中、上下联动的要求，健全四级便民服务网络。拓展“12345”政府公共服务平台功能。推广“一站式”和代理、预约等服务模式。治理庸懒散慢。严格行政监察和问责，坚决纠正不作为、乱作为，促进敢于负责、勇于担当、善于突破、勤于落实。加强公务员教育、培训和管理，增强法治观念和按国际惯例办事的能力。

　　（二）强化机制创新、科学决策。健全依法决策机制，确保决策制度科学、程序正当、过程公开、责任明确。细化重大行政决策程序规定，落实公众参与、专家论证、风险评估、合法性审查、集体讨论决定等法定程序，提高行政决策规范性。推行法律顾问制度。加强规范性文件备案审查。强化行政决策实施后监督、管理和评估，建立重大决策终身责任追究制度及责任倒查机制。

　　（三）推进有效监管、高效执法。坚持宽进严管，切实加强事中事后监管，完善重点领域监管机制，规范中介机构服务行为。深化行政执法体制改革，做到依法有据、裁量公正、执法文明。积极推进综合执法，加大关系群众切身利益问题的行政执法力度。完善行政执法程序和具体操作流程，建立行政裁量权基准制度，严格落实执法责任制，健全行政执法与刑事司法衔接机制。

　　（四）保持公开透明、清正廉洁。巩固党的群众路线教育实践活动成果，自觉践行“三严三实”，严格执行纪律，深入反对“四风”，持之以恒落实中央八项规定和省、市委十项规定，促进为民、务实、清廉。加快推进行政决策、执行、管理、服务和结果等政务公开，全面公开财政预决算和“三公”经费，深化行政权力网上公开透明运行。健全“三解三促”等直接联系服务群众制度，重视社会监督、舆论监督。自觉接受人大及其常委会的法律监督、工作监督和政协的民主监督，认真办好人大代表议案、建议和政协建议案、提案。加强行政权力风险防控，健全政府内控机制，严格“三资”监管。推进审计监督在重点领域全覆盖。全面落实党风廉政建设主体责任，加强惩治和预防腐败体系建设，严格执行廉洁从政各项规定，依法严肃查处违法违纪案件。

　　各位代表，南通正处于新常态下创新转型的重要战略机遇期和黄金发展期。让我们紧密团结在以习近平同志为核心的党中央周围，在省委、省政府和市委的坚强领导下，锐意改革、求真务实，团结拼搏、埋头苦干，为“迈上新台阶、建设新江苏”作出新贡献，谱写好中国梦的南通新篇章！
